# Production Performance and Nutrient Conversion Efficiency of Field Cricket (*Gryllus bimaculatus*) in Mass-Rearing Conditions

**DOI:** 10.3390/ani12172263

**Published:** 2022-09-01

**Authors:** Jamlong Mitchaothai, Nils T. Grabowski, Rachakris Lertpatarakomol, Tassanee Trairatapiwan, Ty Chhay, Sath Keo, Achara Lukkananukool

**Affiliations:** 1 Department of Animal Production Technology and Fisheries, School of Agricultural Technology, King Mongkut’s Institute of Technology Ladkrabang (KMITL), Bangkok 10520, Thailand; 2 Institute for Food Quality and Food Safety, Hannover University for Veterinary Medicine, Foundation (TiHo), 30171 Hanover, Germany; 3 Basic Veterinary and Animal Science Department, Faculty of Veterinary Medicine, Mahanakorn University of Technology (MUT), Bangkok 10530, Thailand; 4 Livestock Development for Community Livelihood (LDC), Phnom Penh P.O. Box 2423, Cambodia; 5 Faculty of Veterinary Medicine, Royal University of Agriculture (RUA), Phnom Penh P.O. Box 2696, Cambodia

**Keywords:** mass rearing, production performance, nutrient conversion efficiency, field cricket

## Abstract

**Simple Summary:**

Farming edible insects such as field crickets (*Gryllus bimaculatus*), called the Mediterranean cricket, is increasingly being adapted for more commercial purposes. Adapting the mass cricket-rearing conditions for field cricket production, we found crickets had a typical growth rate and capacity for conversion of ingested feed into body mass. The efficiency of the deposition of major nutrients (inorganic matter, protein, fat, fiber, and carbohydrate) in the field crickets from the ingested feed is possible to be measured under mass cricket-rearing conditions. The feed intake and mortality rate for cricket rearing should be considered for calculating major nutrient conversion efficiency as increased mortality rate resulted in higher conversion efficiency.

**Abstract:**

Currently, there is an increased interest in mass producing edible insects, e.g., field crickets (*Gryllus bimaculatus*), due to their market value and sustainable development. The current study aimed to measure the production performance of field crickets and to quantify the major nutrient deposition rate using a new approach for a nutrient conversion efficiency calculation for the field crickets under mass-rearing conditions. The field crickets were reared under mass-rearing conditions in the rearing crates and fed with a commercial cricket feed. Measurements for daily feed offered, final body weight, and dead cricket quantity were carried out during the feeding trial period. There were three production rounds with the same procedure for farmed cricket management. The samples of diet, adult crickets, and dead crickets were collected and then analyzed for chemical analysis of macronutrients. The production performance and nutrient conversion efficiency were calculated and then compared with applicable earlier reports for both field and house (*Acheta domesticus*) crickets. The production performance for the studied field crickets under mass-rearing conditions had final a body weight, an average daily gain (ADG), a feed conversion ratio (FCR), and a survival rate of 0.95 g, 23.20 mg/day, 2.94 and 88.51%, respectively. The field crickets had nutrient conversion efficiency for dry matter (DM), ash, crude protein (CP), crude fat (EE), crude fiber (CF), and nitrogen-free extract (NFE) of 13.26, 8.03, 28.95, 88.94, 34.87, and 1.85, respectively, with an adjusted nutrient conversion efficiency of 14.85, 8.99, 32.37, 99.17, 38.95, and 2.10, respectively. Thus, the production of field crickets could be performed under mass-rearing conditions, and the nutrient conversion efficiency for both adjusted and non-adjusted values could be measured.

## 1. Introduction

The global population is expected to reach 9.7 billion by 2050. Alternative food sources for humans and feed for animals are currently being explored to overcome the planet’s finite resources with a sense of sustainable production for food security [1]. Consuming insects (entomophagy) has a long tradition in Southeast Asian countries with relatively widespread use in the region. This tradition has the potential to be used as mini livestock by families, as many insect species can be raised sustainably on agricultural side streams with less of an ecological impact than more typical livestock. On a global level, edible insect farming has just started, bringing the species toward a long process of domestication [2]. As of 2020, commercial insect rearing is growing as an industrial sector across North America and Europe [3], including Southeast Asia [4]. The advantages of insect farming are high nutritional values, low emissions of greenhouse gases, much less land required, and a very high efficiency in terms of food-to-feed conversion when compared with traditional livestock farming [3].

Among the species known to be edible, the insects in the orders Coleoptera, Lepidoptera, Hymenoptera, Orthoptera, and Hemiptera, are commonly consumed [5]. Within the Orthoptera order, crickets are the most consumed insects across the globe [6,7]. Nowadays, there are many publications [2,4,5,8,9,10,11,12,13] related to their nutritive values, properties for improving health, social benefits, and valuable roles in contributing to the economies of many countries and livelihoods.

From the informative report of Kemsawasd et al. [4], Thai farmers have gained more interest in the mass rearing of field (*Gryllus bimaculatus)* and house (*Acheta domesticus*) crickets due to their short production cycle (45–60 days) among edible cricket production and abundant returns. The key difference between these two species of crickets is that *G. bimaculatus* stands out in terms of economic factors (e.g., market value and sustainable development (SDG 12)), while *A. domesticus* has a more acceptable taste (e.g., nutty and a flavor of umami) [4]. However, there were about double lower frequencies of all available reports when comparing *G. bimaculatus* with *A. domesticus* [8]. This would lead to lower information availability on *G. bimaculatus* production performance and related points of view, although more important for the economic impact scale [14]. From the literature review of Kuo and Fisher [8], studies of farmed crickets (Insecta: Orthoptera: Gryllidae spp.) mostly concern insect species, treatment diets, feed measurements, insect measurements, and life stage and duration. The literature review also pointed out that the lack of study standardization is a major problem, such as feed measurements of macro- and micronutrients and cricket measurements, resulting in a difficulty of comparison across studies. In addition, mass rearing seems to be adaptable for both field and house crickets [15,16]. Many studies of edible crickets concerning the production performance and nutrient contents of feed and crickets mainly use small-scale rearing units with a small sub-population of experimental crickets as a model for cricket rearing. Protein conversion efficiency (PCE) is a key metric to determine the ecological footprint of grain-based protein [17]. House cricket production has high PCE compared to the industrial-scale production of conventional livestock, close to the production of carp, egg, and milk, but rather far away from chicken, pork, and beef [17,18]. In the meantime, some reports have used the efficiency of conversion of ingested food (ECI) [6,17,19,20,21] to allow for comparison across studies with more comprehensive and informative data. However, studies are required for small or laboratory scales to quantify production performance and other related feed-nutrient utilization. Most nutritional studies for edible crickets have focused on optimum feed ingredients and resources for rearing crickets, especially protein content. Other nutrients, such as fat and carbohydrates, should receive more attention to understand their relation and possible interaction. In addition, a low survival rate should be considered a criterion to improve the biomass, health, and management of crickets. In the future, there is a trend of industrial or commercial scale for cricket production in accordance with mass-rearing conditions, which is not applicable for performing in a laboratory or on a small scale to quantify the production performance and nutrient conversion efficiency in large-scale cricket production. From a livestock production point of view, feed efficiency represents the cumulative efficiency for conversion from dietary nutrients to get maintenance, lean gain, and lipid accretion, which strongly influences financial returns as closely associated with feed costs [22], and also allows to easily adjust for suitable feed ration for crickets. Additionally, the nutrient conversion efficiencies of feed mirror the environmental performance of the livestock production system [23], including cricket production as mini livestock animals. However, there is little known about the nutrient conversion efficiency for crickets. Therefore, the present study aimed to measure the production performance and to quantify the major nutrient deposition rate using a new approach for the nutrient conversion efficiency calculation for the field crickets under mass-rearing conditions.

## 2. Materials and Methods

### 2.1. Cricket Rearing and Management

To measure the production performance and nutrient conversion efficiency of the field crickets, *G. bimaculatus*, the crickets were reared at a farm facility belonging to the Animal Science Division, Department of Animal Production Technology and Fisheries, School of Agricultural Technology at King Mongkut’s Institute of Technology Ladkrabang, Thailand. Eight rearing cricket crates with a size of 60 cm (width) × 120 cm (length) × 60 cm (height) were made with polycarbonate walls and flooring with a nylon-wire mesh cover, which aimed to allow for ventilation, protection from flies, and prevention of natural predators (such as geckos). Egg board cartons (29.5 cm × 29.5 cm × 4.7 cm) were continuously arranged in a zigzag direction and then arranged into each rearing crate by making a platform for the cartons at around 10 cm above the crate floor. This was to allow floor cleaning, collecting of dead crickets, and prevention of moisture accumulation. Approximately 10–15% of the crate floor area was free from egg cartons, allowing ventilation on the floor of the rearing crates and working convenience. All crates were placed on a shelf with their feet placed in vegetable oil traps to prevent ants and other predators from accessing the cricket eggs and their crickets. Other facilities and equipment required for mass rearing were also prepared, such as plastic bags, a small sweeper, record books, etc. Cricket eggs of the field crickets were purchased from a commercial cricket breeder in Thailand (Somjainuk cricket’s farm, Nakhon Sawan, Thailand). Concerning the mass-rearing system, Parajulee et al. [24] mentioned a mass production of the house crickets of approximately 6000 harvested crickets per rearing unit, with roughly 3000 g for the house crickets (6000 crickets × approximately 0.5 g of cricket body weight). The current study was designed to produce at least 3000 g of field crickets per rearing crate, to be met by the criteria of mass rearing for crickets. Obtained cricket eggs were allocated in approximately equal portions, an average of 5703 eggs per rearing crate estimated from total live crickets/average body weight/survival rate/hatching rate (85% hatching rate assumed from unpublished data) of laying bedding material and egg mixture into each rearing crate and then allowed to hatch from the eggs. Each rearing crate was equipped with a feeding plastic tray with a rough surface, provided on the crate floor and the top of the carton. The feeding regime was *ad libitum* by adding several feeding trays and an amount of feed proportional to the larger size of crickets. The amount of feed offered to the experimental crickets was recorded and a new feed of approximately 600–800 milliliters of water was provided once, twice, and three times a day for the 1st–3rd, 4th–5th, and 6th-week old field crickets, respectively, through approximately 100 cm length and 3.81 cm diameter of PVC pipe (rectangle shape) with 22 holes on its rough surface filled with thread to allow the crickets to sip water through the thread.

To investigate the nutrient conversion efficiency, a commercial cricket feed (Pure Pride^®^; PP feed, the TFMS (Saraburi) Co. Ltd., Saraburi province, Thailand) containing 20.24% crude protein (CP) and 3.22% crude fat was used, which was also used as a dietary treatment in the study of Bawa et al. [25] as Diet I, II, and III containing a commercial cricket feed (PP feed), PP feed replaced by 50% Betagro chicken feed and PP feed supplemented with 100 g dry pulp pumpkin powder/day, respectively. The rather similar environmental condition, feed, and rearing management caused the authors to include it in the current study. Although a preliminary study was performed for field crickets, there is only one replication, in which some aspects were used as guidance for the current study, e.g., survival rate calculation as body weight basis and nutrient composition of dead crickets (Supplementary Table S1). The study of Bawa et al. [25] was used as the main reference data to compare the production performance and for calculating the nutrient conversion efficiency, because the environmental conditions and diet are very similar to the current study (similarity or small difference in Supplementary Table S2). Furthermore, available information for further calculation of the nutrient conversion efficiency was considered a crucial criterion for making comparison possible. The main differences between the current study and the study of Bawa et al. [25] are the cricket species used and the scale of study as 34.28 kg total yield per production round and 2.89 kg total yield per treatment, respectively (Supplementary Table S2), however we tried to apply a way of measuring to be able to compare the results between these two studies. After hatching the nymphs, the Pure Pride cricket feed was offered to the nymphs or crickets throughout the experiment lasting every day, by mixing the leftover feed with the new offered feed. The total feed offered for 8 rearing crates was recorded by weight. During the period of rearing, dead cricket carcasses and parts of cricket bodies were collected and weighed twice a day, after which they were stored with earlier samples of dead crickets until the end of each batch of rearing at −20 °C until further analysis. To reduce the contaminants of dead cricket samples, a mesh was applied to separate the dust or small particles from the dead crickets. When the crickets had reached more than 95% full growth by wings appearing, it was the end of each rearing batch. Around 5–7 days before the end of each batch, a tray with a moisturized mixture of coconut flakes and dust was offered, allowing some female crickets to lay some eggs. The eggs from the crickets were used for the second batch and eggs from the second batch were used for the third batch. One day before harvesting the crickets, 50 males and 50 females were randomly collected from each rearing crate to record the individual final body weight with an analytical balance (Metler Toledo, ML802, Greifensee, Switzerland). Subsequently, the feed was withdrawn at 12 h before harvesting and the left-over feed was measured for weight. At harvesting, all crickets in each crate were weighed and debris or dead crickets were finally collected for the dead cricket sample. After harvesting, all crickets were killed by freezing, and approximately 500 g of crickets were randomly sampled and then stored at −20 °C until further analysis.

### 2.2. Chemical Analysis

Samples of the experimental diet, live crickets, and dead crickets were quantified for dry matter (DM), ash, crude protein (CP), crude fat (EE), crude fiber (CF), and nitrogen-free extract (NFE). The proximate compositions were estimated by following the standard methods recommended by the Association of Official Analytical Chemists (AOAC, 1990). The moisture percentage was calculated by drying the sample in an oven at 60 °C for 3 days. The dried sample was put into a desiccator, allowed to cool and reweighed. The process was repeated until a constant weight was obtained. Crude protein was determined by the Kjeldahl method and the total protein content was calculated as the amount of total N determined multiplied by a nitrogen-to-protein conversion factor of 6.25 for the diet sample. For the samples of live and dead crickets, the conversion factor of 5.0 was used to multiply N contents instead of the conversion factor of 6.25 as an overestimate of protein for field and house crickets [26]. The fat percentage was calculated by drying fat after extraction in a Soxhlet using diethyl ether. Ash percentage was calculated by combusting the samples in a silica crucible placed in a muffle furnace. The percentage of nitrogen-free extract (NFE) or carbohydrate was estimated by the method of difference.

### 2.3. Measured Variables and Calculations

The number of crickets per rearing crate was estimated by dividing the weight of biomass by an average adult body weight. The reported value of 1.36 mg for the first weight after the hatching of *G. bimaculatus* [27] was used as average body weight at the beginning. The preliminary study also showed an average of 1.355 mg body weight for newly hatching crickets. This value was also applied for house crickets in the reference for calculating the beginning weight as well, because the reference, Bawa et al. [25], demonstrated the results of the body weight in the form of only a graph starting at day 1 cricket age, and thus the value was very hard to estimate from the graph. An average daily gain (mg/day) was calculated as total body weight gain (mg)/duration of rearing (day). At the end of each rearing batch, the survival rate was calculated from the weight of live cricket × 100/{(total weight of live crickets) + (2 × total weight of dead crickets)} (more details in Supplementary Table S3), while the report of Bawa et al. [25] used the number of individuals alive at the end of their study. For the feed conversion ratio (FCR), the weight of feed ingested by crickets/weight increase of crickets was calculated on a fresh basis. The efficiency of conversion of ingested food (ECI) is a measure for feed conversion efficiency on a dry matter (DM) basis. ECI (%) is calculated as weight gained × 100/weight of ingested food [28]. This ECI can be called DM conversion efficiency. Protein or nitrogen conversion efficiency was already used to compare within and across reports. To calculate CP conversion efficiency, nitrogen conversion efficiency (N-ECI) was calculated as (insect N-content × insect weight at harvest)/(dietary N-content × feed provided) [6], but CP was calculated (N × 5.0) and used instead of N in this work as the details mentioned earlier. To broaden the use of the ECI concept, other nutrients’ (ash, crude fat, crude fiber, and NFE) conversion efficiencies were calculated in the same way as the CP conversion efficiency. The adjusted nutrient conversion efficiency was calculated as the nutrient conversion efficiency, but nutrient contents in dead crickets were added to nutrient contents of live crickets before making the ratio for the efficiency. For the house crickets, the DM and macronutrient composition was assumed to be equal to the live crickets. This is caused by no available information of DM and nutrient composition of dead crickets, and no significant difference of DM and nutrient composition between live and dead crickets in the current study. The body weight of the dead crickets was assumed as half of the crickets at harvesting. For the half of the body weight at harvesting assumed and applied for calculation, the linear mortality curve (conversion of survival curve) and linear body weight growth curve were assumed from the reports of Parajulee et al. [24] and Bawa et al. [25], respectively. Consequently, the measurable body weight of dead crickets should be half of the dead cricket weight if all died at harvesting (more details in Supplementary Table S3). To compare the nutrient intake and deposition between the field and house crickets, the intake and deposition were calculated as mg of DM and nutrients per individual cricket body weight. Finally, the results from earlier reports with sufficient data support for calculating the adjusted ECI were obtained by selecting the crickets in the experimental groups, offered diets containing 18.9–22.0% CP.

### 2.4. Statistical Analysis

All data were analyzed using IBM SPSS Statistics 28 (IBM, Armonk, NY, USA) to look for differences in the results from the current experiment and the reference. Mean ± standard deviation was expressed where results were appropriate. Differences were tested by Student’s *t*-test.

## 3. Results

### 3.1. Production Performance

The results of production performance are illustrated in Table 1. The production performance for the current study of the field crickets agreed with the earlier reports [19,20,29,30], indicating the success of production under a mass-rearing condition, especially the high survival rate of the current experimental crickets. However, there was a longer duration for development, body weight, and average daily gain when compared to the house crickets fed with a diet containing 18.9–21.9% CP. Higher FCR and lower survival rates were found in the field crickets.

### 3.2. Nutrient Conversion Efficiency

The components of calculation for both ECI and adjusted ECI are shown in Table 2. For the diets used for both the current study and the reference report, they contained almost similar contents of DM, ash, CP, EE and NFE, especially CP and NFE composing the main proportion of nutrients in the diets. The amount of DM and nutrient deposition were expressed as per batch or overall production round, resulting in remarkable differences in actual intake and deposition between these two studies. When the nutrient conversion efficiency was calculated for DM (equal to ECI) and other nutrients, the results showed no difference between the field and the house crickets, except for a higher rate (*p* < 0.05) of conversion for DM after correction by dead crickets and CF both before and after the correction. The statistical analysis (not shown) illustrated no difference (*p* > 0.05) between nutrient conversion efficiency and adjusted nutrient conversion efficiency for both DM and each nutrient. The calculations for the amounts of feed intake and deposition per 1 g of body weight cricket for DM and nutrients help us to make a comparison between the field and the house crickets (Table 3.). From this table, field crickets consumed less (*p* < 0.05) CF than house crickets but deposited more of it (*p* < 0.05). Dead Mediterranean crickets deposited significantly less CP (*p* < 0.05) than house crickets did.

### 3.3. ECI and Adjusted ECI

The adjusted ECI had a higher value compared to the ECI for the current study and the referral report, including other reports about ECI for crickets available at this moment (Table 4). From the results in this table, a lower survival rate resulted in a marked change in the adjusted ECI in each report for both the field and the house cricket species.

## 4. Discussion

For comparisons in the current study, it is likely improper to use results from other studies to compare with the results of the current study. A meta-analysis would be applicable for this kind of comparison. However, it is not the main objective of the current study. Instead, an approach for quantifying the DM and macro-nutrient conversion efficiency applied for the mass rearing of crickets was focused on for this study.

For the production performance, a comparison between different experiments might be improper because production performance is mainly influenced by animal genetics, feed offered, rearing management, and environmental conditions. Among these factors for the comparison in the current study, animal genetics might be the main factor influencing the results of production performance. This might be the fact that other factors had small differences between both studies and the values of the production performance obtained from this study were in accordance with the earlier reports. All relevant values of production performance were used in calculating the DM and macro-nutrient conversion efficiency. Thus, it is implied as important background information for further calculations of the nutrient conversion efficiency. Obtained production performance of the field crickets under mass-rearing conditions was comparable with that of earlier reports performed in both mass-rearing and small-scale experiments, which indicates validation for the production performance values.

There is important data of the field cricket production, which is available for calculation at the present. However, the environmental conditions and the overall management of the crickets, especially the CP content in the diet were comparable, although slight differences in CF contents did occur (Table 2).

The main productive differences between the field and house crickets are the bigger size, shorter duration of rearing, and faster growth rate for field crickets. These are advantages for the field crickets when compared with the house crickets. However, the field crickets had higher FCR and lower survival rates. The latter was also described in the study of Sorjonen et al. [19], who found similar results, especially for the crickets in the control group fed with chicken feed or the commercial feed. Providing an appropriate diet, stock density, and environmental temperature and relative humidity are crucial factors affecting the survival of crickets. These might be explanations for the rather high survival rate of both field and house crickets reared in tropical regions such as Thailand (the current and Bawa et al. [25] study in Table 4) when compared with those reared in western regions of mainly low temperatures. Some insects hibernate when temperatures are low [31] and lower than the developmental threshold [32]. A possible mechanism is that if body temperature exceeds the limits of the cricket’s enzymatic capacity, consequently, the enzymes denature and eventually the cricket die [33,34]. For relative humidity, high relative humidity was beneficial for immature survival, adult longevity and fecundity, and population growth of *Apolygus lucorum* [35], but starvation was found when water was in abundance [36]. From the Cricket Rearing Handbook [15], housing conditions for crickets should maintain temperature range of 28–35 °C and relative humidity of 60–65%, which is almost fitted with the housing conditions of open air barn (temperature of 28.92 ± 1.01 °C and relative humidity of 67.50 ± 2.89%) [25] in central Thailand. In this way, concerning factors that affect the higher mortality rate of the field crickets [37,38] requires more studies, especially the optimal stock density of crickets. For FCR, there are discrepancy results from several reports [7,19,20,30]. The possible explanation for this may be varying focuses of interest for each investigator, in other words, a lack of study standardization for farmed crickets. From the FCR of the current study, there is rather a similar trend of the results compared with those in earlier reports [19,20] which used commercial chicken feeds for crickets as a control or a treatment group, which is comparable to the commercial diet used in the current study. Recording refusal feed might be a cause to reduce the variation of FCR. There are more reports about cricket rearing studies measuring ECI, which were proposed by Waldbauer [28], and others with different diets offered to the crickets. However, the ECI can be calculated as the converse of the FCR in 100%, which means ECI is on a fresh basis. As we have known, the water content in animals and feed ingested by animals will dilute all nutrient contents with different levels for both animal and feed samples. For instance, the ratio of DM in feed: DM in cricket was 89.35:34.93 (2.6:1.0) for the current feeding trial, while it was 93.10:29.56 (3.2:1.0) for the reference report. Therefore, calculating the ratio for ECI on a dry basis would be preferable and no confounding factor for the outcome of the ratio calculation would appear. This study’s calculations for ECI and nutrient conversion efficiency were calculated on a dry basis.

The results in Table 2 were calculated based on biomass or yield of crickets obtained at harvesting and total feed ingested by the crickets for each batch or round of cricket rearing. The remarkably higher values for intake and deposition of DM and other nutrients in the current feeding trial would be caused by larger scale as mass rearing when compared to laboratory scale for the house crickets. This mainly showed the influence of the cricket production scale on feed intake and nutrient deposition, although species difference is a factor affecting the outcome.

However, when nutrient conversion efficiency (as a dry basis) was applied, the difference in values of nutrient conversion efficiency between the field and the house crickets tended to disappear, except for 1.4 times and around 4.0 times deposition of DM and CF, respectively, in the field crickets. This was in agreement with the results of higher ECI (DM conversion efficiency) [19] and higher CF contents for the field crickets compared to the house crickets [39]. The calculation of intake and deposition per g of cricket in Table 3 led to the observation that the field crickets ingested less CF but deposited more CF in the body, indicating higher efficiency for conversion of CF from feed to the CF in the body of the field crickets. This is also probably partly an explanation for higher ECI and adjusted ECI for the field crickets. The CF contents in cricket bodies were contributed from chitin acting as an exoskeleton for crickets [40,41]. The bigger size of the field cricket may imply a higher quantity of exoskeleton required to support bigger body size [42] and faster growth of the field crickets by attempting to deposit CF at a higher rate when compared to the house crickets. From the results in Table 3 and Table 4, it was rather clear that ash, CP, EE, and NFE were deposited on both cricket species proportionally obtained their own nutrients. However, two main factors of differences in cricket species and feed composition used in the comparison might be confounding factors for interpreting the results, because different diets caused different contents and different cricket species had different capacities to exploit the resources of nutrients, which required further study to elucidate it. At least, the current study has shown that ECI could be calculated under both mass rearing and small-scale rearing. When considering the values of nutrient conversion efficiency (both adjusted and non-adjusted), there was the lowest deposition rate (1–2%) for NFE and the highest deposition rate (60–99%) for EE. It might be hypothesized that both field and house crickets can convert carbohydrates (NFE) into lipids [13,43]. As we have known, feeds containing high carbohydrate contributes to increased lipid deposition both in human and livestock animals. However, there is still a need for further study to prove this hypothesis for crickets. This high rate of fat deposition into the cricket bodies would lead to being an appropriate food as ketogenic diets for reducing obesity [10].

Concerning the calculation of adjusted ECI and nutrient conversion efficiency (Table 2 and Table 4), the adjusted values were higher than the non-adjusted ones. This would be the result of the contents of dead crickets included in the calculation. From Table 4, when the mortality rate of crickets was high, there was a trend for a positively proportional increase in ECI. In fact, the death of very small instars of crickets is very hard to be completely collected. However, these very small sizes also had little impact on the whole volume of dead crickets. From the point of view of livestock production, science research and also aquatic animal farming [44], the loss of experimental group animals will affect estimating the actual feed ingested. Thus, adjusted the ECI should be useful to gain more insight into nutrient metabolism and utilization. However, both the non-adjusted values and survival rates of crickets are still important to be quantified to be considered together. Under mass cricket-rearing conditions, the measurement of the survival rate from the weight of dead crickets should be applicable and more feasible for the involved staff. In addition, other factors that may affect the contents of dead crickets should be investigated further, e.g., instar-related compositional differences.

## 5. Conclusions

The production of field crickets (*G. bimaculatus*) could be performed under mass-rearing conditions without major adverse effects on the production performance. Recording feed intake and collecting dead crickets during the period of cricket rearing are key activities to monitor cricket performance, allowing possibilities to calculate the value of nutrient conversion efficiency for both adjusted and non-adjusted values under mass cricket rearing. This probably helps to get more insight into nutrient metabolism and utilization of crickets, at least by allowing comparable values across studies as the higher adjusted conversion efficiency related to a higher mortality rate of crickets.

## Figures and Tables

**Table 1:** Production performance for the experimental field in mass-rearing conditions and referral house cricket under laboratory-scale conditions.

Data Source	Cricket Species	Diet ^a^	Duration of Rearing (days)	Body Weight (g)	Average Daily Gain (mg/day)	Feed Intake per Animals (g)	Feed Conversion Ratio; FCR (Feed: Gain)	Survival Rate (%)
Current study	Field cricket	Batch I–III	41.0 ± 1.0	0.95 ± 0.17	23.20 ± 4.49	2.78 ± 0.39	2.94 ± 0.12	88.51 ± 1.01
Bawa et al., 2020 [25]	House cricket	Diet I–III	49.0 ± 0.0	0.48 ± 0.07	9.73 ± 1.36	0.78 ± 0.04	1.58 ± 0.13	96.00 ± 1.00
		*p*-Value	**	*	*	*	***	***

^a^ Batch I–III are the same diet containing 20.2% CP, while diet I, II, and III contain 21.9%, 18.9%, and 19.6% CP, respectively; other nutrient composition is provided in Table 2. *, **, *** Significant difference between the field and house crickets at *p* < 0.05, *p* < 0.01, and *p* < 0.001, respectively.

**Table 2:** Nutrient conversion efficiency calculation from biomass, dead cricket carcasses and survival rate of the experimental field crickets in mass-rearing condition in comparison to the referral house cricket under laboratory-scale conditions.

Item	DM	Ash	CP	EE	CF	NFE
Diet composition (%)						
Field cricket (experiment)	89.35	6.03	20.24	3.22	3.88	66.62
House cricket (Reference)	93.10 ± 5.14	6.03 ± 0.58	20.13 ± 1.57	2.33 ± 1.01	5.87 ± 0.25	65.60 ± 0.79
DM and nutrient intake (kg/batch)						
Field cricket (experiment)	83.74 ± 5.44	5.05 ± 0.33	16.95 ± 1.10	2.70 ± 0.18	3.25 ± 0.21	55.78 ± 3.62
House cricket (Reference)	4.21 ± 0.26	0.26 ± 0.04	0.85 ± 0.06	0.10 ± 0.05	0.25 ± 0.00	2.76 ± 0.15
Live cricket composition (%)						
Field cricket (experiment)	34.93 ± 3.15	3.66 ± 0.31	44.28 ± 3.88	21.51 ± 4.71	10.25 ± 1.19	9.23 ± 1.25
House cricket (Reference)	29.56 ± 0.32	4.50 ± 0.10	59.47 ± 2.30	13.70 ± 5.25	5.57 ± 1.62	7.40 ± 2.55
DM and nutrient deposition in live crickets (kg/batch)						
Field cricket (experiment)	11.13 ± 1.31	0.40 ± 0.03	4.90 ± 3.73	2.42 ± 0.76	1.13 ± 0.07	1.04 ± 0.25
House cricket (Reference)	0.42 ± 0.06	0.02 ± 0.00	0.25 ± 0.04	0.06 ± 0.01	0.02 ± 0.01	0.03 ± 0.01
Dead cricket composition (%)						
Field cricket (experiment)	31.99 ± 3.57	3.70 ± 0.25	43.99 ± 3.73	20.22 ± 4.42	10.09 ± 1.35	10.65 ± 1.10
House cricket (Reference)	29.56 ± 0.32	4.50 ± 0.10	59.47 ± 2.30	13.70 ± 5.25	5.57 ± 1.62	7.40 ± 2.55
DM and nutrient deposition in dead crickets (kg/batch)						
Field cricket (Experiment)	1.342 ± 0.347	0.049 ± 0.010	0.583 ± 0.116	0.281 ± 0.127	0.133 ± 0.027	0.145 ± 0.050
House cricket (Reference) ^a^	0.009 ± 0.001	0.000 ± 0.000	0.005 ± 0.001	0.001 ± 0.001	0.000 ± 0.000	0.001 ± 0.000
Nutrient conversion efficiency (%)						
Field cricket (Experiment)	13.26 ± 0.73	8.03 ± 0.47	28.95 ± 1.98	88.94 ± 23.06	34.87 ± 2.45	1.85 ± 0.34
House cricket (Reference)	10.11 ± 1.94	7.70 ± 2.18	29.92 ± 5.98	60.59 ± 13.69	9.45 ± 2.84	1.17 ± 0.54
*p*-Value	ns	ns	ns	ns	***	ns
Adjusted nutrient conversion efficiency (%)						
Field cricket (Experiment)	14.85 ± 1.04	8.99 ± 0.48	32.37 ± 2.10	99.17 ± 27.11	38.95 ± 2.64	2.10 ± 0.41
House cricket (Reference) ^a^	10.32 ± 1.94	7.85 ± 2.19	30.52 ± 5.94	61.81 ± 13.74	9.64 ± 2.86	1.20 ± 0.55
*p*-Value	*	ns	ns	ns	***	ns

^a^ Dead cricket composition was assumed as the same composition as live crickets. *, *** Significant difference between the field and house crickets at *p* < 0.05 and *p* < 0.001, respectively. ns = Not significant (*p* > 0.05).

**Table 3:** Intake and deposition in live and dead crickets as weight proportion for mg of DM and nutrient per g of crickets.

Item	DM	Ash	CP	EE	CF	NFE
DM and nutrient intake (mg/g)						
Field cricket (Experiment)	2,328.26 ± 75.84	140.37 ± 4.57	471.33 ± 15.35	75.08 ± 2.45	90.39 ± 2.94	1,551.08 ± 50.52
House cricket (Reference)	2,821.22 ± 561.29	172.35 ± 51.87	564.63 ± 89.75	69.05 ± 43.05	164.60 ± 25.50	1,849.38 ± 358.33
*p*-Value	0.2665	0.3975	0.2109	0.8309	0.0357	0.2851
DM and nutrient deposition (mg/g)						
Field cricket (Experiment)	349.33 ± 31.48	12.75 ± 1.13	154.37 ± 15.68	75.58 ± 20.10	35.56 ± 1.11	32.49 ± 7.03
House cricket (Reference)	295.60 ± 3.20	13.30 ± 0.40	175.79 ± 7.30	40.45 ± 15.39	16.42 ± 4.65	21.91 ± 7.76
*p*-Value	ns	ns	ns	ns	*	ns
DM and nutrient deposition in dead crickets (mg/g)						
Field cricket (Experiment)	319.95 ± 35.66	11.79 ± 0.88	140.04 ± 10.13	65.65 ± 20.75	32.05 ± 2.78	34.29 ± 6.90
House cricket (Reference) ^a^	295.60 ± 3.20	13.30 ± 0.40	175.79 ± 7.30	40.45 ± 15.39	16.42 ± 4.65	21.91 ± 7.76
*p*-Value	ns	ns	**	ns	*	ns

^a^ Dead cricket composition was assumed as the same composition as live crickets. *, ** Significant difference between the field and house crickets at *p* < 0.05 and *p* < 0.01, respectively. ns = Not significant (*p* > 0.05).

**Table 4:** The efficiency of conversion of ingested food (ECI) corrected by dead crickets used for the current study and the literature review.

Source	Field Cricket			House Cricket		
	Survival Rate	ECI	Adjusted ECI	Survival Rate	ECI	Adjusted ECI
Current study	88.51	13.26	14.85	na	na	na
Dobermann et al. [20]	55.00	10.86 *	15.30 *	na	na	na
Jansom et al. [30]	27.15	10.50	16.46	na	na	na
Bawa et al. [25]	na	na	na	96.00	10.11	10.32
Oonincx et al. [6]	na	na	na	55.00	11.66	16.42
Sorjonen et al. [19]	47.33	15.94	25.28	81.17	5.78	6.49

* As usual results available, 90% DM of ingested cricket feed and 30% DM cricket body were assumed for calculation of both ECI and adjusted ECI, na = data not available.

## Data Availability

Not applicable.

## Supplementary Materials

The following supporting information can be downloaded at: https://www.mdpi.com/article/10.3390/ani12172263/s1, Table S1: Results of a preliminary study; Table S2: Similarity and difference between the current study and the study of Bawa et al. (2020); Table S3: Methods of calculating survival rate of crickets.
